# Pharmacomicrobiomics: exploiting the drug-microbiota interactions in anticancer therapies

**DOI:** 10.1186/s40168-018-0483-7

**Published:** 2018-05-22

**Authors:** Concetta Panebianco, Angelo Andriulli, Valerio Pazienza

**Affiliations:** Division of Gastroenterology, IRCCS “Casa Sollievo della Sofferenza” Hospital, Viale dei Cappuccini, 1, 71013 San Giovanni Rotondo, FG Italy

**Keywords:** Microbiota, Cancer, Metagenomics, Chemotherapy, Immunotherapy

## Abstract

Cancer is a major health burden worldwide, and despite continuous advances in medical therapies, resistance to standard drugs and adverse effects still represent an important cause of therapeutic failure. There is a growing evidence that gut bacteria can affect the response to chemo- and immunotherapeutic drugs by modulating either efficacy or toxicity. Moreover, intratumor bacteria have been shown to modulate chemotherapy response. At the same time, anticancer treatments themselves significantly affect the microbiota composition, thus disrupting homeostasis and exacerbating discomfort to the patient. Here, we review the existing knowledge concerning the role of the microbiota in mediating chemo- and immunotherapy efficacy and toxicity and the ability of these therapeutic options to trigger dysbiotic condition contributing to the severity of side effects. In addition, we discuss the use of probiotics, prebiotics, synbiotics, postbiotics, and antibiotics as emerging strategies for manipulating the microbiota in order to improve therapeutic outcome or at least ensure patients a better quality of life all along of anticancer treatments.

## Background

The human intestine is home to about 3.8 × 10^13^ microorganisms reaching the weight of almost 1.8 kg, collectively named as gut microbiota, which are now widely recognized to maintain host physiology and health by exerting fundamental functions, spanning from metabolic to immunomodulatory properties [1, 2]. To fulfill these tasks, the microbiota establishes an equilibrium with the host, a condition referred to as eubiosis. Perturbations of this balance, resulting in altered microbial ecology, have been associated with different diseases, including cancer [3, 4]. It is noteworthy that microbial dysbiosis not only contributes to cancer pathogenesis and progression but also influences the therapeutic outcome, this latter function being essentially related to the microbial ability to metabolize drugs and xenobiotics and to modulate host inflammation and immune responses [5]. Anticancer drugs are specifically designed with the aim to be effective in the treatment of malignancies, but being generally toxic also for normal cells, their use carries on numerous side effects, some of which are life-threatening. The adverse effects may require a reduction of the drug dosage or the change of drug regimen to make the treatment tolerable to the patient. Another major inconvenient in anticancer treatments is the development of drug resistance, which is recognized as the primary cause of failure of chemotherapeutic treatment of most human tumors [6]. This failure can be partly explained by host genetic factors, but it is becoming clear that other aspects are involved. In recent years, efforts have been devoted to develop therapeutic approaches with more specificity for cancer cells and a lesser toxicity for the host. In this setting, immunotherapy has introduced a paradigm shift in oncology, with the use of drugs targeting immune cells rather than cancer cells, aimed at stimulating the antitumor immune response of patients [7].

Both in the case of chemotherapy and immunotherapy, resident microorganisms are known to interfere with host-targeted therapy directly or indirectly with three main clinical outcomes: (i) facilitate drug efficacy, (ii) abrogate and compromise anticancer effects, and (iii) mediate toxicity [8]. Conversely, it is also apparent that cancer itself and anticancer therapies affect the microbiota profile in patients.

In recent years, the interaction between microbiota and anticancer drugs is drawing a growing interest, as well as the setup of interventions aimed at shaping microbiota to optimize drug efficacy and reduce side effects. In this regard, it has been proposed the concomitant administration of probiotics, prebiotics, synbiotics, postbiotics, or antibiotics with anticancer therapy in order to rebalance the gut microbiota [9]. Probiotics are defined as viable microbial species providing health benefits, the most studied being *Lactobacilli* and *Bifidobacteria*, while prebiotics are non-digestible compounds (mainly fibers) stimulating the growth/activity of beneficial bacteria [9–11]. Probiotics and prebiotics may represent a way to restore commensal microorganisms suppressed by anticancer therapy and a healthy gut environment. They can be combined in the so called synbiotics, formulations in which the prebiotic compounds selectively favor the growth of probiotic organisms yielding a synergistic effect [10, 11]. Furthermore, the use of postbiotics, that is to say nonviable microbial products or metabolites with biological activities [9, 10, 12], can also mimic the beneficial effects of probiotics administration. Postbiotics, such as short-chain fatty acids butyrate, acetate, and propionate, may provide to the host the benefits that are generally assured by an healthy and balanced microbiota. Another approach to selectively target undesired bacteria by killing them or arresting their growth is the administration of very selective antibiotics, which should only be administered to reverse the overgrowth of bacteria dangerous for patient’s health [9, 10]. Better elucidating the interplay between chemotherapy, immunotherapy, and microbiota may uncover new therapeutic targets and innovative integrated approaches to improve the clinical management of cancer patients.

## Microorganisms influence chemotherapy response and toxicity

**Fig. 1:**
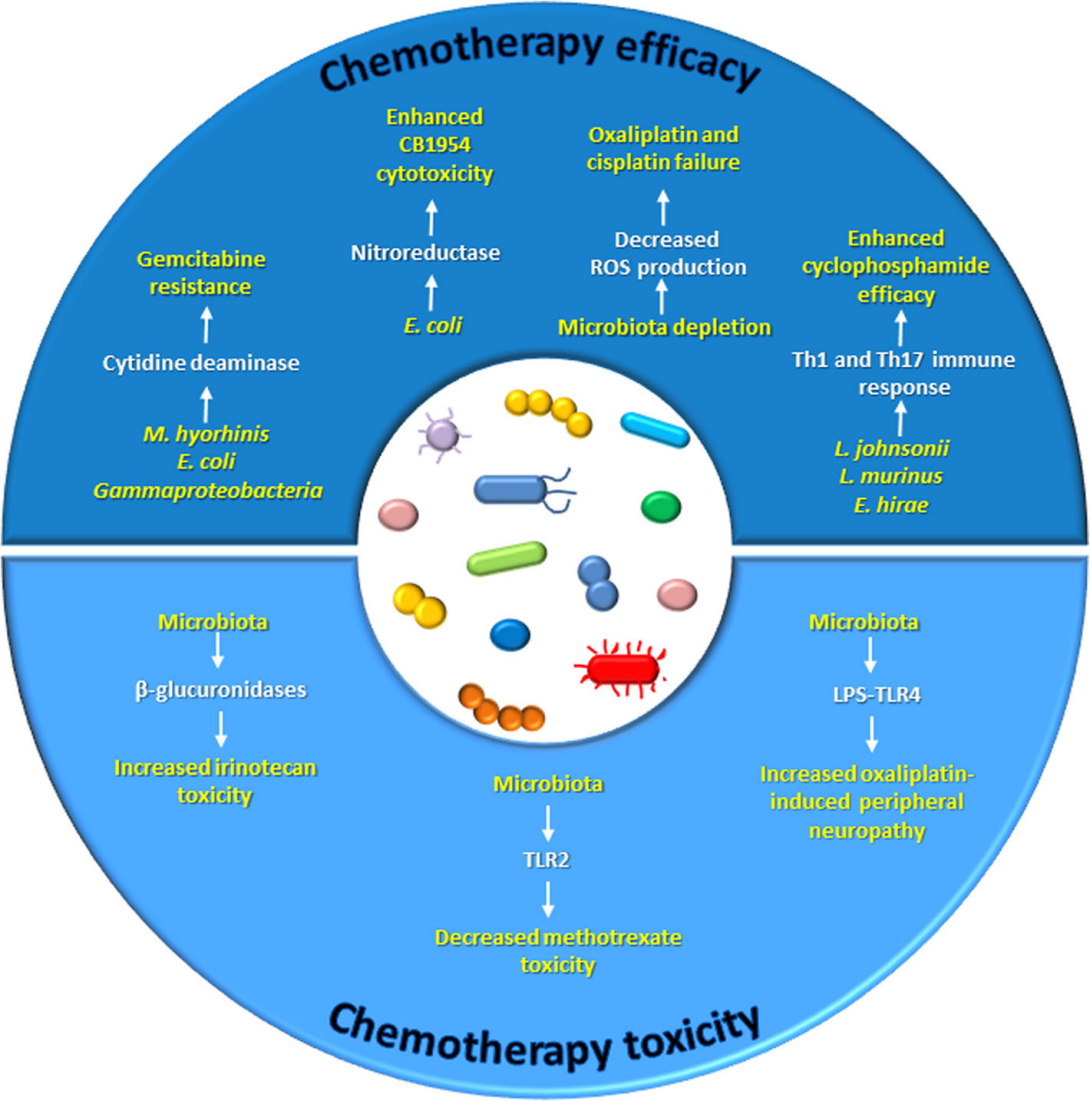
The microbiota modulates chemotherapy efficacy and toxicity

As represented in Fig. 1, both gut microbiota and intratumor bacteria can modulate chemotherapy efficacy and mediate its toxic effects. Several lines of evidence exist about the presence of certain bacteria in tumor tissues and their ability to modulate chemotherapeutic drug response [13–15]. Infections from *Mycoplasma* species, especially *Mycoplasma hyorhinis*, and the presence of this bacterium in tumor tissues have been documented in several types of cancers [16, 17]. It is known that these microorganisms express nucleoside analog-catabolizing enzymes, which could impair drug efficiency [15, 17]. Indeed, mice subcutaneously injected with *M. hyorhinis*-infected colon cancer cells exhibited gemcitabine resistance, and this effect was due to gemcitabine (2′,2′-difluorodeoxycytidine) deamination to its inactive metabolite 2′,2′-difluorodeoxyuridine [13]. A number of bacterial species other than *Mycoplasma*, mainly belonging to *Gammaproteobacteria*, were found to confer gemcitabine resistance, which was dependent on the expression of a bacterial long form of the enzyme cytidine deaminase. Interestingly, in a colon cancer mouse model, gemcitabine resistance caused by intratumor *Gammaproteobacteria* was reversed by co-administration of the antibiotic ciprofloxacin, thus supporting the role of these bacteria in failed drug response [13].

Similarly, Lehouritis et al. provided in vitro evidence that bacteria may influence the value of a chemotherapy regimen, decreasing the activity of certain drugs while improving the efficacy of some others, through enzymatic biotransformation and chemical modification of the pharmacological molecules. In the same study, in vivo confirmation was provided that *Escherichia coli* impairs gemcitabine efficacy as demonstrated by increased tumor volume and reduced survival, while increasing the cytotoxicity of the drug CB 1954 [14], a pro-drug whose activation is strongly enhanced by *E. coli* nitroreductase activity [18].

The involvement of the microbiota in the response to chemotherapy was also verified for platinum compounds [19, 20], whose mechanism of action relies not only on the formation of platinum-DNA adducts which block DNA replication and stimulate ROS production and oxidative damage but also on their ability to stimulate immune response.

Iida and colleagues showed that mice bearing subcutaneous EL4 lymphoma treated with a cocktail of antibiotics displayed significantly reduced cancer regression and survival upon oxaliplatin therapy. Similar results were observed when using germ-free mice, antibiotic-treated mice with subcutaneous colon carcinoma, and antibiotic-treated mice with EL4 lymphoma receiving cisplatin. In this study, the cause of therapy failure was identified in a decreased microbiota-dependent ROS production [20]. Consistent with this study, Gui et al. observed an increase in tumor size and a consequent decrease in survival rate in a mouse model of lung cancer receiving cisplatin combined with antibiotics as compared to animals receiving cisplatin alone. Conversely, mice administered with cisplatin combined with *Lactobacillus* bacteria showed a better response to therapy. The observed effects were associated to the modulation of the expression of VEGFA, BAX, and CDKN1B genes in the tumor and on bacterial enhancement of the T cell immunity [19].

At the crossroad between chemotherapy and immunotherapy is the clinical use of cyclophosphamide (CTX), an alkylating drug whose anticancer functions also rely on stimulation of anticancer immunity. Viaud et al. demonstrated that CTX treatment in tumor-bearing mice caused the translocation of a set of Gram-positive species (*Lactobacillus johnsonii*, *Lactobacillus murinus*, and *Enterococcus hirae*) into mesenteric lymph nodes and the spleen, where they were instrumental in the stimulation a Th1 and Th17 immune response. Germ-free mice or animals treated with an antibiotic against Gram-positive bacteria failed to generate this response and proved resistant to the drug [21]. In a subsequent study, the authors demonstrated that oral gavage with *E. hirae* restored the response to CTX in tumor-bearing antibiotic-treated mice [22].

Beside the impact on chemotherapy efficacy, bacteria may also modulate drug toxicity and side effects. An exemplification of this concept is the pro-drug irinotecan, mainly employed in the treatment of advanced colorectal cancer, which becomes activated to SN-38 metabolite upon removal of a piperidino moiety by a carboxylesterase, subsequently glucuronidated to its inactive form SN-38G in the liver and eliminated through biliary excretion. Once in the intestine, bacterial β-glucuronidases reconvert SN-38G to SN-38, thus restoring the drug activity which is responsible also of a severe intestinal toxicity [23, 24]. Wallace et al. demonstrated that, in comparison with animals receiving irinotecan alone, co-administration of irinotecan with a selective inhibitor of bacterial β-glucuronidase prevented either colonic damage or the appearance of diarrhea [23]. Recently, based on the greater or lesser ability to reactivate SN-38G to SN-38, 20 fecal samples collected from 20 healthy subject humans who underwent treatment with irinotecan were subgrouped into either high or low metabolizers: compared to low metabolizers, the microbiomes of high metabolizers contained significantly higher levels of three types of microbial β-glucuronidases [25]. In addition, in rats treated with irinotecan, a correlation between changes in fecal microbiota and drug-induced gastrointestinal toxicity was uncovered. A significant decrease in microbial diversity and an increase in *Fusobacteria* and *Proteobacteria* were observed, which have been all associated with intestinal inflammation [26].

As for irinotecan, also for methotrexate (MTX), gastrointestinal damage is a major side effect of therapy and the microbiota may be involved. In this case, however, a positive role for bacteria against toxicity emerged, since genetic knockout of TLR2 or microbiota depletion by antibiotics in mice resulted in a more severe intestinal mucositis. In myeloid cells, TLR2 stimulation increased the expression and activity of the multidrug resistance pump ABCB1/MDR1 suggesting drug efflux as a mechanism for limiting inflammation and toxicity [27].

Further evidence of the link between bacteria and chemotherapy toxicity was provided by Shen et al., who demonstrated a role for gut microbiota in oxaliplatin-induced peripheral neuropathy: transient eradication of mouse gut microbiota, achieved through the administration of antibiotics, reduced oxaliplatin-induced pain [28]. Similar results were obtained in germ-free mice, but the benefit burned out following restoration of a gut microbiota. Although the precise mechanism remains to be clarified, the interrelationship between microbial LPS-TLR4 on macrophage cells seems to be determinant of the hyperalgesia [28].

## Microorganisms influence immunotherapy response and toxicity

A key concept in cancer immunology is that tumor cells develop strategies to escape from immunosurveillance, which normally would recognize and eliminate them [29]. The idea of boosting the immune system to fight cancer led to the development of the immunotherapy as a new and alternative tool in the clinical oncology [7].

**Fig. 2:**
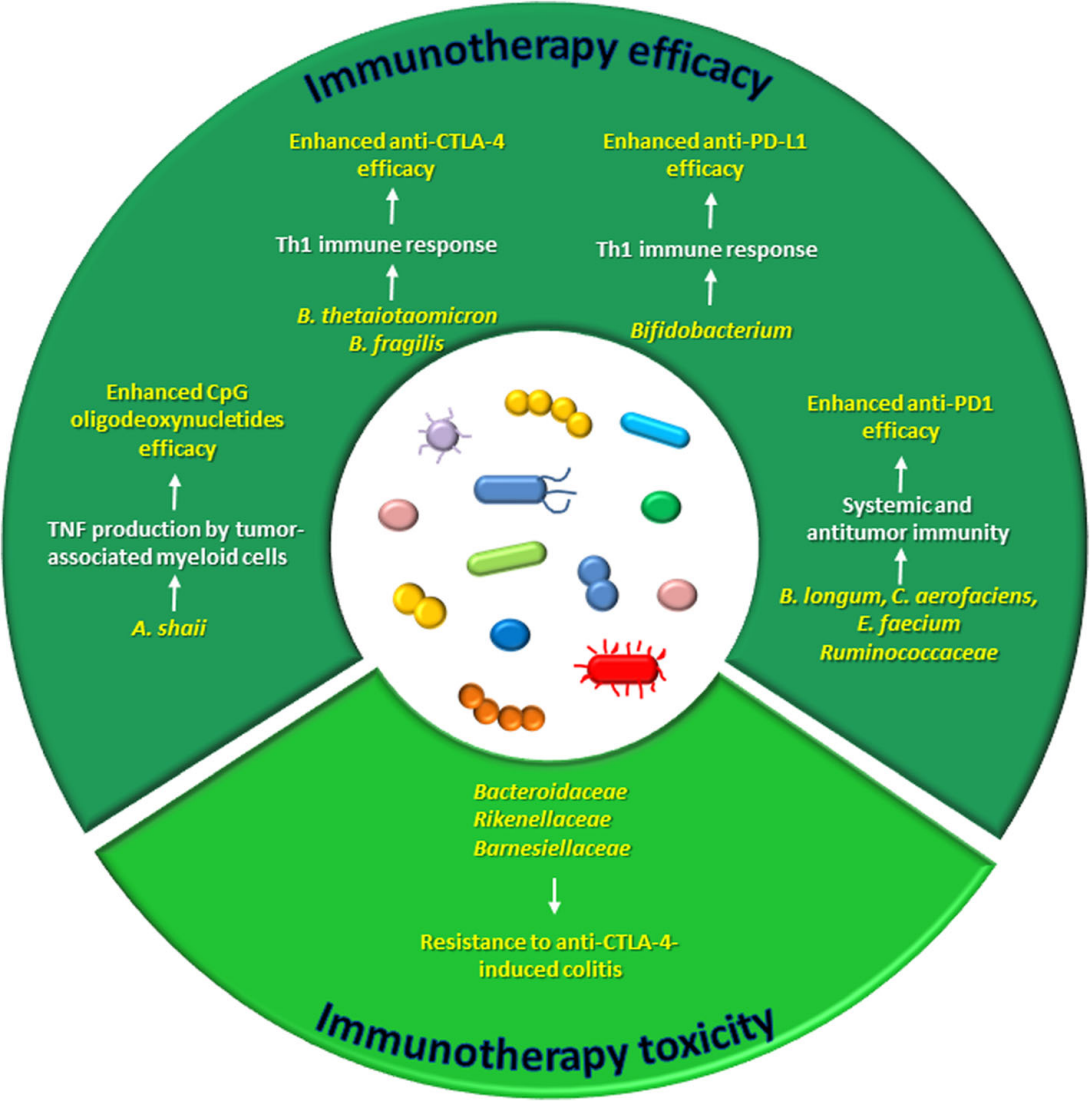
The microbiota modulates immunotherapy efficacy and toxicity

As microbiota strongly modulates inflammation and immunity, it is plausible that alterations in microbiota composition can affect immunotherapy response (Fig. 2).

CpG oligodeoxynucleotides are synthetic molecules containing unmethylated CG dinucleotides and mimicking bacterial DNA, which have shown potent immunostimulatory properties and antitumor activity in several kinds of cancer [30].

In the work by Iida et al., mice bearing EL4 lymphoma, MC38 colon carcinoma and B16 melanoma were given intratumor CpG oligodeoxynucleotides in combination with an antibody against the interleukin-10 receptor: the combined treatment delayed tumor growth and prolonged survival, benefits which were related to enhanced tumor necrosis factor (TNF) production from tumor-associated myeloid cells and to the activation of cytotoxic CD8 T cells. Of relevance, the efficacy of this approach was reduced in mice whose microbiota was depleted by antibiotics and in germ-free mice. A further investigation led to identify *Alistipes shaii* as a bacterial species positively associated with TNF production by tumor-associated myeloid cells. Oral administration of this microorganism to antibiotic-treated mice restored TNF production [20].

Another strategy in anticancer immunotherapy is represented by the blockade of immune checkpoints, such as cytotoxic T-lymphocyte-associated antigen 4 (CTLA-4) and programmed death 1 (PD-1), which are negative regulators of T cell proliferation and functions [29].

CTLA-4, also known as CD152, is a T cell surface receptor which, once activated upon binding to its ligands (CD80 or CD86) expressed on antigen presenting cells, triggers an inhibitory signal in early activated T cell [29]. Ipilimumab is a monoclonal antibody against CTLA-4 approved for clinical use in the treatment of metastatic melanoma, whose efficacy was found to depend on gut microbiota. Indeed, in mouse models of MCA205 sarcoma, Ret melanoma, and MC38 colon carcinoma, ipilimumab response was abolished in germ-free animals or in those treated with broad-spectrum antibiotics. Specifically, *Bacteroides thetaiotaomicron* and *Bacteroides fragilis* were identified as responsible of CTLA-4 blockade and ipilimumab efficacy through the induction of a Th1 immune response [31]. In addition to mediate therapy response, gut microbiota is also involved in ipilimumab-induced colitis [32, 33]. An analysis of fecal microbiota performed on melanoma patients undergoing anti-CTLA-4 treatment revealed differences between subjects prone and subjects resistant to colitis: bacteria within the *Bacteroidetes* phylum in general, and specifically, the families of *Bacteroidaceae*, *Rikenellaceae*, and *Barnesiellaceae*, were more abundant in patients who did not manifest adverse events. Moreover, a shortage of the bacterial genetic pathways involved in polyamine transport and B vitamin biosynthesis was found associated with an increased risk of developing ipilimumab-induced colitis [32]. More recently, administration of the antibiotic vancomycin to mice with colitis and treated with anti-CTLA-4 resulted in a more severe manifestation of the intestinal disease, while administration of *Bifidobacterium* alleviated the symptoms [33].

The other immune checkpoint repressing T cell response, namely the axis programmed cell death protein 1 (PD-1) and its ligand (PD-L1), intervenes in later stages of immune response than CTLA-4. PD-1 is expressed on the surface of activated T cells while PD-L1 is expressed on tumor cells and on antigen presenting cells: their binding causes T cell inactivation [34]. A number of monoclonal antibodies against PD-1 and PD-L1 have been employed in the immunotherapy of several types of cancer. Sivan et al. demonstrated that gut microbiota mediates the response to anti-PD-L1 therapy, since they observed different outcomes in terms of melanoma growth and antitumor immune response in two populations of genetically similar mice housed in different facilities and harboring different microbiota. *Bifidobacterium* emerged as strongly associated with T cell response, and consistently, oral administration of a cocktail of *Bifidobacterium* species combined with an anti-PD-L1 antibody nearly abolished the melanoma growth [35]. Three recent studies demonstrated the impact of gut microbiota on the clinical response to anti-PD1 immunotherapy [36–38]. Matson et al. found that an abundance of *Bifidobacterium longum*, *Collinsella aerofaciens*, and *Enterococcus faecium* was associated with anti-PD1 efficacy in metastatic melanoma patients [37], while Gopalakrishnan et al. observed higher microbial diversity and increased *Ruminococcaceae* levels in responding melanoma patients relative to non-responders, together with an enhanced systemic and antitumor immunity [36]. Fecal microbiota transplantation (FMT) from responding patients to germ-free animals boosted response to treatment [36]. Finally, Routy et al. analyzed the microbiota of patients with non-small cell lung cancer and renal cell carcinoma and found fecal levels of *Akkermansia muciniphila* directly correlated with efficacy anti-PD1 therapy. Unlike FMT from non-responders, FMT from responding patients to germ-free or antibiotic-treated mice improved PD-1 blockade efficacy. However, oral administration of *A. muciniphila* after FMT from non-responders restored response to immunotherapy [38]. The last three studies reported different bacteria associated with a better response to the blockade of the same immune check point. As commented also by Jobin [39], this discrepancy could be explained by the different cancer types, the different genetic background of the cohorts of patients enrolled, and the interpopulation variability of the microbiota composition. Moreover, it should be taken into account that differences among the studies may arise, also, from the different metagenomic approaches used (16S sequencing [36], whole genome shotgun sequencing [38], or an integration of the two [37]) and consequently different bioinformatic and biostatistic analysis tools used for microorganism identification/association.

## Chemotherapy shapes microbiota

Pathological disease states or surgical therapies create a condition of dysbiosis [40], and chemotherapeutics further exacerbate the dysbiotic state, potentially leading to the occurrence of adverse events [8].

Before the advent of the next-generation sequencing approaches, some reports already existed about the impact of chemotherapy on gut microbiota. In 2003, by using culture-based methods, it was demonstrated that 5-fluorouracil (5-FU) administration to rats perturbs oral and gastrointestinal microbiota composition and localization. As for intestinal bacteria, an overall increase of Gram-negative anaerobes was found after 5-FU treatment, and an increased translocation to mesenteric lymph nodes [41]. Few years later, changes in the composition of microbiota in different portions of the gastrointestinal tract were detected with culture methods in rats upon 5-FU administration, and a qRT-PCR-based quantification of the fecal microbiota revealed an increase in *Clostridium* species, *Staphylococcus* species and *E. coli* abundance, and a decrease in *Lactobacillus* species and *Bacteroides* species [42].

In 2009, the effect of chemotherapy treatment on gut microbiota in pediatric patients with acute myeloid leukemia by using polymerase chain reaction/denaturing gradient gel electrophoresis and fluorescent in situ hybridization to analyze and quantify bacterial populations was reported [43]: during the chemotherapeutic treatment, the number of bacteria in fecal samples was 100-fold lower than that in healthy control samples, which resulted in a lower diversity of intestinal microbiota. *Bacteroides* species, *Clostridium cluster XIVa*, *Faecalibacterium prausnitzii*, and *Bifidobacterium* species (which are among the predominant intestinal anaerobic bacteria) decreased 3000–6000-fold in samples during treatment, compared with the healthy control samples. The number of pathogenic enterococci was significantly higher while the number of streptococci decreased in patient samples. The authors also examined, in in vitro experiments, the direct effects of chemotherapy on bacterial growth and found that etoposide and daunorubicin inhibited the growth of *Clostridium* species, *Streptococcus mitis*, *Bifidobacterium animalis*, and *Lactobacillus acidophilus* but not the growth of enterococci or *E. coli.* Overall, their findings suggest that selective killing of commensal anaerobes following the administration of chemotherapeutics allows the expansion of potentially pathogenic microbes [43].

Similar to 5-FU, also, CTX was shown to alter composition and localization of intestinal bacteria [21, 44]. Viaud et al. observed a decrease in the phylum *Firmicutes* distributed within *Clostridium* cluster *XIVa*, *Roseburia*, *Lachnospiraceae*, and *Coprococcus* and a reduction in lactobacilli and enterococci in the mucosa of mice exposed to CTX. In addition, CTX treatment caused a significant translocation of several Gram-positive species into mesenteric lymph nodes and spleen, due to increased intestinal permeability [21]. Consistently, Yang et al. revealed that CTX administration to mice increased intestinal permeability and the count of potentially pathogenic bacteria such as *E. coli*, *Pseudomonas*, *Enterobacteriaceae*, and enterococci [44]. In a subsequent study comparing control mice with CTX-treated mice, several substantial differences were reported in the composition of fecal microbiota. In detail, at the phylum level, the *Firmicutes*/*Bacteroidetes* ratio increased from 0.50 to 0.90, together with *Bacteroidetes* significantly reduced, *Actinobacteria* significantly increased, and *Verrucomicrobia* disappeared in treated mice compared to controls. At the class level, CTX treatment caused an increase in *Bacteroidia* and *Alphaproteobacteria* and a decrease in *Bacilli*, *Clostridia*, *Coriobacteriia*, and *Mollicutes.* At the family level, *Lachnospiraceae*, *Coriobacteriaceae*, *Lactobacillaceae*, and *Staphylococcaceae* were more abundant; *Prevotellaceae*, *S24-7*, *Alcaligenaceae*, and *Rhodospirillaceae* were less represented; and *Streptococcaceae* were absent in CTX-receiving mice [45].

In a colon cancer-bearing rat model, irinotecan increased the abundance of *Clostridium* cluster XI and *Enterobacteriaceae* which are generally low concentrated in healthy humans and rodents and to which belong several bacteria inducing diarrhea, including *Peptoclostridium difficile* [46].

The gut microbiota profile of patients with non-Hodgkin’s lymphoma was analyzed before and after a 5-day myeloablative chemotherapy regimen with high-dose carmustine, etoposide, aracytine, and melphalan: a significant drop in *Firmicutes* and *Actinobacteria* abundance and a significant increase in *Proteobacteria* when compared to samples collected before chemotherapy were documented [47].

A similar trend was observed in our recent paper [48] describing the influence of gemcitabine therapy on microbiota profile of pancreatic cancer xenografted mice. *Firmicutes* and *Bacteroidetes* were under-represented in the gut of gemcitabine-receiving mice, whose bacterial profile was shifted in favor of two other phyla, *Proteobacteria* (mainly *E. coli*) and *Verrucomicrobia* (mainly *A. muciniphila*), which are generally minor residents of gut microbiota [48, 49]. At lower taxonomic levels, the bacteria belonging to the *Bacteroidales* order were approximately halved in gemcitabine-treated mice as compared to control mice. Similarly, the relative abundance of the *Lachnospiraceae* and of the *Ruminococcaceae* families was reduced following chemotherapy. Furthermore, at the species level, *A. muciniphila* and *E. coli* significantly increased while *B. acidifaciens* decreased upon gemcitabine treatment. Remarkably, *L. animalis*, was detectable in minor amounts in treated animals while *P. difficile*, which was not detected in control mice, increased in the gemcitabine group. The overall alteration observed in microbiota composition upon gemcitabine treatment is suggestive of a pro-inflammatory bacterial selection [48].

All these studies are consonant with the assumption that chemotherapy treatments disrupt intestinal microbiota homeostasis (see Table 1), allowing the overgrowth of pathogenic bacteria which, by exacerbating or perpetuating the intestinal injury induced by chemotherapy, contribute to the development of adverse events [50].

**Table 1:** Influence of chemotherapeutic treatments on intestinal microbiota profiles

Chemotherapeutic treatment	Microbiota modifications	Reference
5-Fluorouracil	Increase in Gram-negative anaerobes Increased translocation to mesenteric lymph nodes	[41]
	Increase in *Clostridium* spp., *Staphylococcus* spp., and *Escherichia coli* and decrease in *Lactobacillus* spp*.* and *Bacteroides* spp*.*	[42]
Cycles I and II: high-dose cytarabine, daunorubicin, and etoposide; cycle III: amsacrine, high-dose cytarabine, and etoposide; cycle IV: mitoxantrone and high-dose cytarabine	Lower total number and diversity of intestinal bacteria; decrease in *Bacteroides* spp., *Clostridium cluster XIVa*, *Faecalibacterium prausnitzii*, and *Bifidobacterium* spp.; increase in pathogenic enterococci and decrease in streptococci	[43]
Cyclophosphamide	Decrease in *Clostridium cluster XIVa*, *Roseburia*, *Lachnospiraceae*, *Coprococcus*, lactobacilli, and enterococci Increased translocation of Gram-positive species to mesenteric lymph nodes and spleen	[21]
	Increased *Escherichia coli*, *Pseudomonas*, *Enterobacteriaceae* and enterococci Increased *Firmicutes*/*Bacteroidetes* ratio	[44]
	Increased *Actinobacteria*, *Bacteroidia*, *Alphaproteobacteria*, *Lachnospiraceae*, *Coriobacteriaceae*, *Lactobacillaceae*, and *Staphylococcaceae*; decreased *Bacteroidetes*, *Bacilli*, *Clostridia*, *Coriobacteriia*, *Mollicutes*, *Prevotellaceae*, *S24-7*, *Alcaligenaceae*, and *Rhodospirillaceae*; disappeared *Verrucomicrobia* and *Streptococcaceae*	[45]
Irinotecan	Increased *Clostridium cluster XI* (including *Peptoclostridium difficile*) and *Enterobacteriaceae*	[46]
High-dose carmustine, etoposide, aracytine, and melphalan	Increased *Proteobacteria*, decreased *Firmicutes* and *Actinobacteria*	[47]
Gemcitabine	Increased *Proteobacteria*, *Verrucomicrobia*, *Akkermansia muciniphila*, *Escherichia coli* and *Peptoclostridium difficile*; decreased *Firmicutes*, *Bacteroidetes*, *Bacteroidales*, *Lachnospiraceae*, *Ruminococcaceae*, *Bacteroides acidifaciens* and *Lactobacillus animalis*	[48]

## Immunotherapy shapes microbiota

Compared to chemotherapy, much less evidence has been provided concerning a role for immunotherapy in the modulation of gut microbiota. A study from Vetizou et al. reported that the anti-CTLA-4 treatment with ipilimumab alters the microbiota composition in the human and mouse gut. In particular, in the feces of mice subjected to treatment, *Bacteroidales* and *Burkholderiales* decreased and *Clostridiales* increased. In patients with metastatic melanoma, the microbiota profile revealed three main groups dominated by *Prevotella* and *Alloprevotella* (cluster A) and distinct species of *Bacteroides* (cluster B and C). After ipilimumab therapy, however, a shift of patients from cluster B to cluster C was observed, suggesting that anti-CTLA-4 therapy favors the dominance of selected *Bacteroides* species [31].

## Manipulating gut microbiota to improve anticancer therapeutic outcome

Nowadays, it is well known that a number of factors, spanning from diet and lifestyle to environment, can considerably influence the composition of gut microbiota [51].

**Fig. 3:**
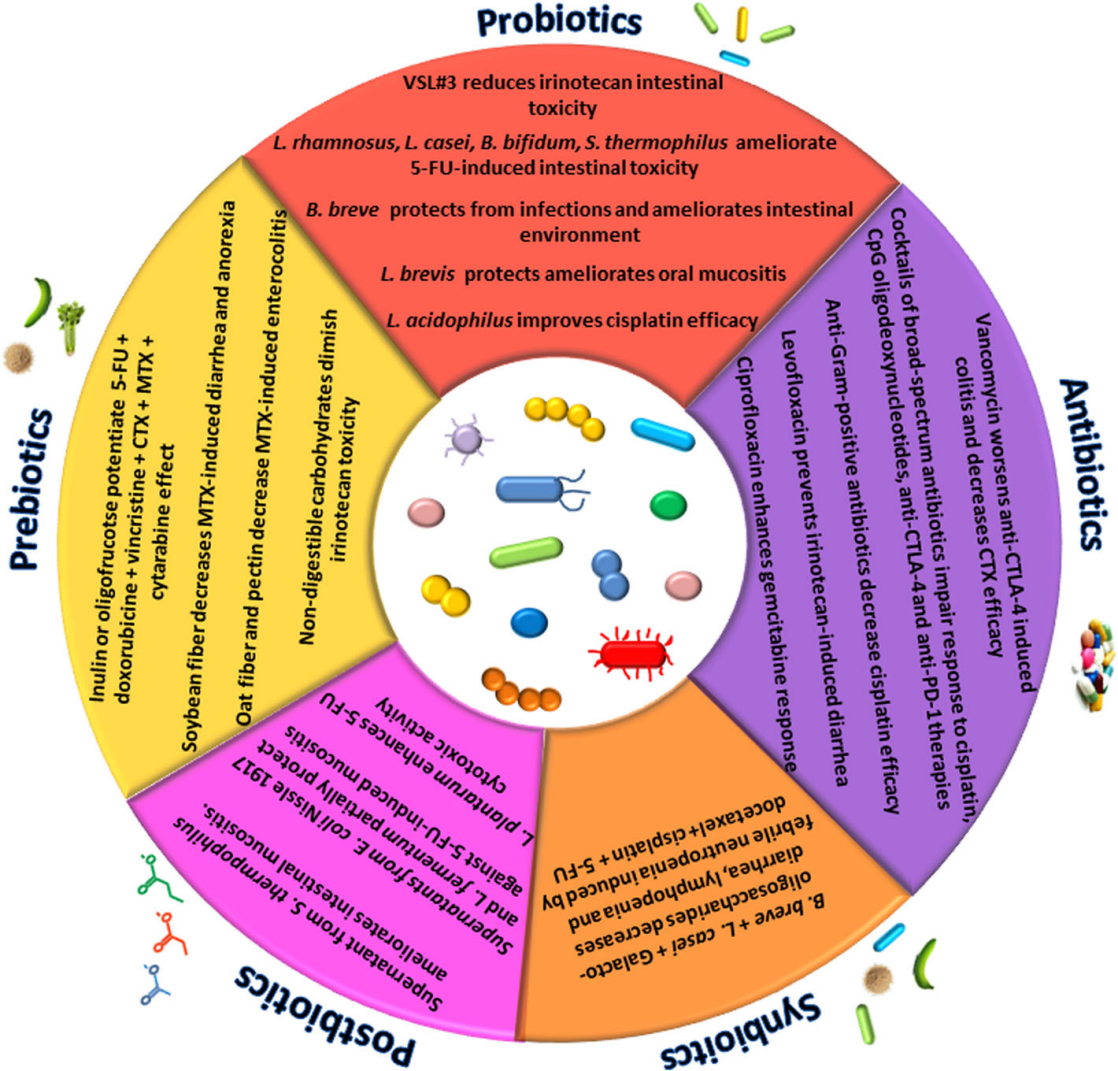
“Biotic” strategies to modulate microbiota and the outcome of anticancer therapies

The data discussed above undoubtedly place the microbiota in a relevant position in the setting of the anticancer drug response. Hence, strategies aimed at manipulating gut microflora (summarized in Fig. 3) are increasingly recognized as a valid tool to improve therapeutic outcome. Below, we review the main targeted interventions that are being proposed to correct dysbiosis, thus helping the clinical management of cancer.

### Probiotics

According to the definition elaborated by FAO and WHO, the term probiotics refers to “live microorganisms which, when administered in adequate amounts, confer a health benefit on the host”. Most of probiotics are lactic acid-producing bacteria, especially belonging to the genera *Lactobacillus* and *Bifidobacterium*; other genera such as *Streptococcus*, *Bacillus*, and *Enterococcus* are also used, with some concerns regarding their safety, since some strains of these genera are potentially pathogenic. Moreover, *Saccharomyces* yeasts have been also used as probiotics [52, 53].

Several mechanisms are supposed to be responsible for the beneficial effects exerted by probiotics: interaction with colonocytes and maintenance of the intestinal barrier, production of antimicrobial factors (such as H_2_O_2_, bacteriocins, defensins, short-chain fatty acids) which suppress pathogen growth, competition for adhesion and nutrients with potentially harmful microorganisms, degradation of toxins, regulation of enzymatic activities in the colon, and activation of the immune response [19, 54–56].

While extensive literature exists concerning the application of probiotics in the prevention of several diseases, including cancer, there is still lack of clarity about their use in support to therapy. A certain number of studies investigated the potential of probiotics to ameliorate toxicity of chemotherapies. Mego et al. performed two small clinical studies in which *Enterococcus faecium* M-74 was administered to few patients with testicular cancer or leukemia undergoing chemotherapy, but no preventive effect against drug-induced febrile neutropenia was observed [57, 58]. Similarly, the use of the probiotic strains *Lactobacillus fermentum* BR11, *Lactobacillus rhamnosus* GG, and *Bifidobacterium lactis* BB12 administered individually to rats receiving 5-FU offered no protection against chemotherapy-related intestinal mucositis [59]. These studies, however, do not exclude the utility of probiotics as adjunctive treatment to chemotherapy, since several factors including bacterial strains and combination thereof, dosage, and duration of administration may explain lack of efficacy. A substantial number of reports, indeed, attest the usefulness of probiotics in association to chemotherapy. The probiotic mixture VSL#3 (which contains *Streptococcus thermophilus*, *Bifidobacterium breve*, *Bifidobacterium longum*, *Bifidobacterium infantis*, *Lactobacillus acidophilus*, *Lactobacillus plantarum*, *Lactobacillus paracasei*, and *Lactobacillus delbrueckii* subsp. bulgaricus) proved to be effective in reducing weight loss and diarrhea induced by irinotecan in rats [60]. Diarrhea was also reduced in patients with colon cancer treated with 5-FU and supplemented with *L. rhamnosus* GG [61]. In line with this result, *Lactobacillus casei* variety rhamnosus and *Bifidobacterium bifidum* ameliorated intestinal mucositis in mice receiving 5-FU [62, 63] and *Streptococcus thermophylus* TH-4 showed similar effects in 5-FU treated rats [64].

In patients affected by different pediatric tumors and immunocompromised because of chemotherapy, *Bifidobacterium breve* strain Yakult was shown to protect from infections and to ameliorate intestinal environment as demonstrated by the concentrations of total organic acids, which were maintained most of the time at the normal level, and subsequently maintained the pH below 7.0. [65].

Probiotics have been shown to relieve also extra-intestinal symptoms of chemotherapy: *Lactobacillus brevis* CD2 resulted promising in ameliorating oral mucositis in patients receiving chemotherapy for head and neck cancer or for hematopoietic stem cell transplantation [66, 67].

The studies presented above demonstrate a role for probiotics in alleviating chemotherapy side effects, which itself represents a remarkable result as it means improving quality of life for patients. Moreover, it should be kept in mind that reducing toxicity may also mean increasing drug efficacy, by avoiding the need to lower drug dosage.

Probiotics administration, however, has also been reported to improve chemotherapy and immunotherapy outcome. Lewis lung cancer xenograft mice treated with cisplatin and fed with *L. acidophilus* solution via the orogastric route displayed a decreased tumor volume together with an extended animal survival as compared to cisplatin treatment alone [19].

*A. muciniphila* administration boosted anti-PD-1 efficacy in antibiotic-treated mice [38] and *Bifidobacterium* administration to mice with melanoma improved response to anti-PD-L1 therapy, nearly abolishing tumor outgrowth [35].

### Prebiotics

As stated above, diet considerably influences gut microbiota composition. In particular, prebiotics are defined as non-viable food components that benefit the host’s health by selectively promoting the growth/activity of one or few microorganisms in the colon [55, 68]. Prebiotics are mainly represented by fibers, that is to say carbohydrates which arrive undigested into the large bowel where they are fermented by commensal bacteria. This fermentation results in the production of short-chain fatty acids (SCFA) which lowers the intestinal pH, sustaining the growth of gut friendly bacteria such as *Lactobacillus* and *Bifidobacterium* [69]. Resistant starch (RS) is one of the most studied prebiotic with the ability to promote the growth of bacteria involved in the production of butyrate. The latter is a well-known postbiotic with anticancer and anti-inflammatory activity [70]. It has been demonstrated that RS retards tumor growth in pancreatic cancer xenograft mice and in parallel shapes microbiota favoring the anti-inflammatory microorganisms while decreasing the pro-inflammatory ones [71].

Most of literature describes a cancer-preventing action of prebiotics; nonetheless, some papers report a supportive effect to chemotherapy, either in terms of therapeutic efficacy or diminished toxicity. Taper et al. tested the effect of administering inulin or oligofructose to mice with transplantable liver tumor treated with subtherapeutic doses of six chemotherapeutics (namely 5-FU, doxorubicin, vincristine, CTX, MTX, cytarabine), observing a potentiation of drug effects in terms of increased life span [72, 73]. More recently, a chemical conjugate of inulin with doxorubicin was produced and tested in vitro on colon cancer cells, displaying the same or improved cytotoxic response at lower doses than doxorubicin alone [74].

Different papers investigated the effects of prebiotics on MTX-induced toxicity in rats: soybean fiber was shown to reduce diarrhea and anorexia [75], and oat fiber [76] and pectin [77] were reported to reduce the severity of MTX-related enterocolitis. Furthermore, diets enriched in non-digestible carbohydrates (isomalto-oligosaccharides, resistant starch, fructo-oligosaccharide, or inulin) administered to rats with colon cancer were found associated to a diminished irinotecan toxicity; although no correlation with specific bacterial taxa emerged, the amelioration observed was correlated with an increase in butyrate production [78].

### Synbiotics

The word synbiotics was coined to mean a combination of probiotics and prebiotics with synergistic effect compared to the sum of the two agents. In a synbiotic formulation, the prebiotic element(s) should selectively promote the growth/activity of the probiotics microorganism(s) [55]. The application of synbiotics to support anticancer therapy has been little investigated so far.

A combination of the probiotic *Lactobacillus fermentum* BR11 with the prebiotics fructo-oligosaccharide was tested on intestinal mucositis caused by 5-FU administration in rats, but the synbiotic failed to provide any further amelioration than the probiotic alone [79].

Conversely, a positive response was obtained by Motoori et al. from the use of synbiotics in esophageal cancer patients receiving neoadjuvant chemotherapy. In detail, a mixture containing *B. breve* strain Yakult, *L. casei* strain Shirota, and galacto-oligosaccharides was compared with the probiotic *Streptococcus faecalis* alone (control group). The synbiotic decreased the severity of diarrhea and lymphopenia and the occurrence of febrile neutropenia caused by the docetaxel, cisplatin, and 5-FU chemotherapy regimen, in comparison to control. The amelioration of symptoms was attributed to changes in gut microbiota composition, since a reduction in harmful and an increase in beneficial bacteria were observed in subjects receiving the synbiotic in respect to control patients [80].

### Postbiotics

Not only viable microorganisms but also their soluble products and metabolites, known as postbiotics, are endowed with biological activities which can benefit the host [9, 12]. The most representative example of postbiotics is certainly the SCFA produced by carbohydrates fermentation.

It has been observed that the culture supernatants of certain probiotics maintain the same effectiveness of alive bacteria, so that postbiotics are in some cases considered a valid and safer alternative to taking viable microorganisms [81].

Some reports of postbiotics efficacy exist in the context of anticancer therapy, too. In the aforementioned study testing *S. thermophilus* TH-4 on 5-FU-related mucositis, the probiotic supernatant showed to inhibit intestinal crypt fission, as did live microorganism [64]. Likewise, supernatants from *E. coli* Nissle 1917 and *L. fermentum* BR11 were shown to partially protect rat gut from 5-FU-induced mucositis [82]. Furthermore, an in vitro study performed on colon cancer cells provided evidence that supernatant from *Lactobacillus plantarum* enhanced 5-FU cytotoxic activity, as demonstrated by increased apoptosis, reduced survival of cancer cells, and inhibition of stemness features [83]. The latter study suggests a potential use of postbiotics in increasing efficacy of chemotherapy beside in alleviating its adverse effects.

### Antibiotics

Antibiotic intake is obviously a factor deeply affecting the composition of microbiota and, as reported in some papers discussed above, a factor able to condition the therapeutic outcome of anticancer treatments. Geller et al. demonstrated that bacteria from tumor microenvironment may contribute to gemcitabine resistance producing a form of the enzyme cytidine deaminase inactivating the drug. Mice with colon cancer treated with gemcitabine plus the antibiotic ciprofloxacin showed enhanced drug response than mice receiving gemcitabine alone [13]. Further, a positive contribution of the antibiotic levofloxacin on irinotecan side effects was observed in patients with metastatic colorectal cancer, by preventing the occurrence of diarrhea [84].

On the other hand, several studies pointed out the negative impact of antibiotics administration on immuno- and chemotherapy efficacy. Depletion of microbiota with an antibiotic cocktail of vancomycin, ampicillin, and neomycin in lung cancer mice treated with cisplatin resulted in increased tumor burden and reduced survival compared to cisplatin therapy alone [19]. A cocktail of the broad-spectrum antibiotics ampicillin, colistin, and streptomycin, as well as the beta-lactam imipenem alone, were found to compromise the anticancer effect of the anti-CTLA-4 therapy in mouse models of sarcoma, melanoma, and colon cancer [31]. Vancomycin pre-treatment worsened the colitis provoked by CTLA-4 blockade in mice [33]. It was recently demonstrated that antibiotics administration compromises the clinical benefits of PD-1-based immunotherapy both in mice and in humans. Indeed, in RET melanoma and MC-205 sarcoma mouse models treated with an anti-PD-1 antibody, a 14-day therapy with the ampicillin, colistin, and streptomycin cocktail results in increased tumor volume and reduced overall survival compared to mice not taking antibiotics. Furthermore, within a subset of patients with non-small cell lung cancer, renal cell carcinoma, or urothelial cancer treated with anti-PD1/PD-L1 therapy, those who were prescribed antibiotics (mainly beta-lactams, fluoroquinolones or macrolides) prior to or concomitant with the first immunotherapy injection showed significantly shorter progression-free survival and overall survival relative when compared to antibiotic-free patients [38, 85]. Similarly, in a cohort of metastatic renal cell carcinoma patients receiving PD-1/PD-L1-based immunotherapy, antibiotic (above all beta-lactams and fluoroquinolones) users had lower objective response rate and progression-free survival relative to non-users [86].

An antibiotic cocktail of vancomycin, imipenem, and neomycin was shown to impair the efficacy of CpG oligodeoxynucleotide immunotherapy in mice with colon cancer and with melanoma and also to reduce response to oxaliplatin-based chemotherapy in mice with lymphoma [20]. Viaud et al. demonstrated that pre-treating mice subjected to CTX with the anti-Gram-positive antibiotic vancomycin failed to activate the anticancer immune response necessary for the chemotherapy effectiveness [21]. With the aim of translating to humans the findings by these last two studies, Pflug et al. recruited a population of patients with chronic lymphocytic leukemia treated with CTX and a population of patients with relapsed lymphoma treated with cisplatin and investigated the impact of anti-Gram-positive antibiotics on clinical outcome. In both populations, the administration of antibiotics was independently associated with reduced progression-free survival and overall survival [87].

## Conclusions

There is a growing body of evidence linking microbiota to the success of anticancer therapies in a bidirectional way, meaning that these two factors can strongly affect and modulate each other. This has led to the concept of “pharmacomicrobiomics” as a new discipline exploring the interactions between drugs and microbes [8, 88].

In order to improve the therapeutic outcome and alleviate drug adverse effects, a number of approaches to selectively manipulate microbiota have been suggested, including administration of probiotics, prebiotics, synbiotics, postbiotics, and antibiotics in support of conventional treatments (Fig. 3). The adoption of these strategies, however, deserves some considerations. First of all, it should be kept in mind that many of the studies here reviewed have been performed in animal models: despite similarities in phylum composition, substantial differences in lower taxa exist between human and murine microbiota; that is why, further investigations are needed before these findings can be translated to the clinical setting. Moreover, concerning the manipulation of microbiota by probiotic administration, some questions have been raised regarding the safety of this approach using viable microorganisms. In particular, the main concerns about probiotic safety concern the risk of infections such as bacteremia or endocarditis, the potential to give toxic or immunological effects, the potential of antibiotic-resistance horizontal transfer between probiotics, and commensal bacteria [89, 90]. Attention should be paid also when administering antibiotics since undesired and non-specific depletion of the microflora could be caused, unless a very selective drug is used. Not least, inter-individual variability in microbiota composition is also a factor to be considered. For instance, in the human gut microbiota, three main enterotypes have been identified based on the abundance of the three bacterial genera *Bacteroides*, *Prevotella*, and *Ruminococcus*, which might explain different responses to drugs [91]. Exploring the individual microbial profile would be a useful step to set up personalized strategies of microbiota manipulation.

Despite these considerations to be taken into account, the results collected so far about the improvement of cancer therapy through microbiota manipulation are exciting and very encouraging. Devoting more efforts in better elucidating the complex network of interactions between drugs, microorganisms and host may pave a new path in the field of clinical management of cancer.

## Abbreviations

5-FU: 5-Fluorouracil
CTLA-4: Cytotoxic T-lymphocyte-associated antigen 4
CTX: Cyclophosphamide
FMT: Fecal microbiota transplantation
MTX: Methotrexate
PD-1: Programmed death 1
SCFA: Short-chain fatty acids
TNF: Tumor necrosis factor
